# A survey of the experiences of delivering physiotherapy services through telerehabilitation during the COVID-19 pandemic

**DOI:** 10.3389/fresc.2024.1486801

**Published:** 2024-10-24

**Authors:** Tzu-Hsuan Peng, Janice J. Eng, Anne Harris, Catherine Le Cornu Levett, Jennifer Yao, Amy Schneeberg, Courtney L. Pollock

**Affiliations:** ^1^ Department of Physical Therapy, University of British Columbia, Vancouver, BC, Canada; ^2^ Centre for Aging SMART at Vancouver Coastal Health, Vancouver, BC, Canada; ^3^ GF Strong Rehabilitation Centre, Vancouver, BC, Canada

**Keywords:** telerehabilitation, physiotherapy, clinicians experience, COVID-19 pandemic, telehealth

## Abstract

**Introduction:**

Physiotherapy services have been typically provided in-person since the profession usually involves a therapist providing hands-on assessment and treatments. The COVID-19 pandemic provided an opportunity to study physiotherapists’ adaptation to telerehabilitation (phone or videoconference).

**Objective:**

This study aimed: (1) to explore how physiotherapists adapted to the transition to delivering telerehabilitation, (2) to assess physiotherapists’ perceptions of implementing telerehabilitation, and (3) to identify the challenges and facilitators of delivering telerehabilitation.

**Methods:**

This study used an online survey distributed to physiotherapists within a large Canadian health authority. Closed-ended questions were analyzed with descriptive statistics.

**Results:**

Seventy-five physiotherapists responded and data were collected. Compared prior to the pandemic to time during the pandemic, the use of a phone for delivering physiotherapy increased from 24.0% to 73.3% of physiotherapists while videoconference increased from 5.3% to 77.3%. Overall, the physiotherapists found videoconference to be a more effective delivery method than phone. Less than half felt that they could use videoconference to effectively treat pain (49.3%), upper extremity function (40.0%) or strength/range of motion (48.0%). Only 29.3% felt that they could effectively treat walking balance or mobility by videoconference. Technical barriers were identified with client comfort with the equipment reported by 90.7% of physiotherapists and positioning of the webcam by 76.0% of physiotherapists. A large proportion of physiotherapists agreed that they would continue the practice of telerehabilitation via phone (54.7%) and videoconference (68.0%).

**Conclusion:**

The pandemic resulted in a dramatic shift to telerehabilitation for a profession that typically provides hands-on assessments and treatments. While there was increased uptake of telerehabilitation, many physiotherapists questioned their effectiveness using telerehabilitation to undertake activities that traditionally involve manual treatments or hands-on guidance/supervision. However, physiotherapists were committed to continuing telerehabilitation to meet patients’ needs after the pandemic.

## 1 Introduction

Due to the outbreak of coronavirus disease 2019 (COVID-19) pandemic, many health institutions reduced their services, especially for outpatient and community services (1). Some services were transitioned to telerehabilitation with phone or videoconference technology. While rehabilitation services such as speech therapy or psychology may lend themselves well to virtual sessions, delivery of physiotherapy services over virtual platforms can pose substantial challenges (2, 3). Physiotherapists (PTs) deliver rehabilitation services that typically provide physical assessment and treatment with clients with a broad range of impairments and often utilize specialized equipment (e.g., treadmills, parallel bars, electric plinths, electrical modalities) and hands-on guidance/supervision.

While some PTs may have used videoconference to deliver rehabilitation services in the past, this was primarily to deliver education or follow-up care (4). The abrupt closure of outpatient services and cancellation of home visits resulted in PTs having to adapt their practice to telerehabilitation during the pandemic (5). Although options for the provision of telerehabilitation had been previously available, the uptake of videoconference was limited prior to the pandemic. The pandemic provided an opportunity to understand the experience of PTs with implementing telerehabilitation services across a broad range of clinical settings and areas of physiotherapy practice.

While other studies have reported an increase in the use of phone and videoconference over the pandemic for rehabilitation clinicians, including PTs (6–9), our study is unique in exploring the impact of telerehabilitation practices unique to PTs (e.g., pain treatment, range of motion/strength, balance training, gait training). Furthermore, our study examined the responses of a whole public health authority, providing care to one of the largest regions in Canada.

This study aimed to investigate the experience of PTs in telerehabilitation (phone and videoconference) for rehabilitation services during the pandemic. The purpose was to: (1) quantify the change in practice of PTs in adapting to the use of telerehabilitation services; (2) assess perceptions of PTs of their ability to utilize telerehabilitation services with their clients; and (3) describe barriers and facilitators to implementation of telerehabilitation services. This study will help to improve the implementation of delivering physiotherapy services remotely.

## 2 Methods

### 2.1 Study design and participants

A cross-sectional study design was used and data were collected via an online self-administered survey. Approval for this study's protocol was obtained from the university's research ethics board and the local health authority. We included individuals who worked in any facility of Vancouver Coastal Health as a practicing PT, and had delivered telerehabilitation physiotherapy sessions during the pandemic. Vancouver Coastal Health is a large health authority that is responsible for the delivery of hospital and community services for over a million people in the Vancouver region. There are approximately 400 PTs that work within this health authority with about 150 PTs providing outpatient services. From the onset of COVID-19 (March, 2020), PTs across Vancouver Coastal Health were mandated to prioritize in-person therapy at a limit of approximately 20% of their caseload.

### 2.2 Survey instrument

Because there were no suitable existing data collection tools identified, an original survey was developed by the research team, using the online software tool Qualtrics (secure and licensed) with close-ended and open-ended questions. The survey was reviewed by 2 academic PTs, a clinical practice leader in PT, a front-line physician, a front-line PT, a statistician, and an occupational therapist. The survey was piloted on 3 PTs and their feedback was integrated. The survey addressed issues of patient safety, meeting rehabilitation goals, outcome measurement, caregiver involvement, technology challenges, burden of work, as well as barriers and facilitators with delivering physiotherapy services over phone or videoconference. The survey link was distributed by email to all PTs who worked in Vancouver Coastal Health providing outpatient therapy. After providing informed consent to participate in the study, participants completed the survey online. The data was collected from November 19th, 2020 to January 15th, 2021, which was the third wave of outbreak in the province of British Columbia. The reminders were sent on weekly basis with a total of 4 reminders sent. The survey is available in the online Supplementary Materials.

### 2.3 Data analysis

Data were downloaded from Qualtrics Survey and processed in a Microsoft Excel spreadsheet. Descriptive statistics were calculated. In presenting the data from the 5-point Likert scale, “Strongly agreed” and “Agree” were consolidated into an “Agree” category, and ‘Strongly Disagree’ and “Disagree’ were consolidated into a “Disagree” category.

## 3 Results

### 3.1 Characteristics of the participants

There were 77 people who completed the survey. Two of the responses were completed by physiotherapy assistants and were excluded from the analysis. The participants are described in Table 1. Of the participants, 81.3% were female. The primary setting of respondents was in outpatient services (54.7%), community health (32.0%), and inpatient or acute services (13.3%). PTs were working primarily in orthopaedics (37.3%), neurological rehabilitation (22.7%) or with mixed caseloads (34.7%). Before the pandemic, the majority of respondents did not have experience in delivering telerehabilitation over phone or video, but majority of respondents reported offering telerehabilitation during the pandemic. The use of a phone increased from 24.0% to 73.3% of PTs while the use of videoconference increased from 5.3% to 77.3%.

**Table 1:** Demographics information of the study population (*n* = 75).

Variables	Categories	*n*	%
Age	21–30	12	16.0
	31–40	22	29.3
	41–50	25	33.3
	51–60	11	14.7
	61–70	5	6.7
Gender	Male	13	17.3
	Female	61	81.3
	Prefer not to answer	1	1.3
Primary work field	Acute care	9	12.0
	Outpatient	41	54.7
	Community	24	32.0
	Inpatient rehabilitation	1	1.3
Practice field	Neurological rehabilitation	17	22.7
	Orthopaedics	28	37.3
	Mixed/general caseload	26	34.7
	Cardio-respiratory and cardiac/pulmonary rehabilitation	4	5.3
Years spent in current area of practice	0–5 years	41	54.7
	6–10 years	13	17.3
	11–15 years	11	14.7
	16–20 years	5	6.7
	21–25 years	2	2.7
	> 25 years	3	4.0
Previous experience with telerehabilitation	Over phone	18	24.0
	Via video	4	5.3
Currently offer physiotherapy:	In-person	72	96.0
	Over phone	55	73.3
	Via video	58	77.3
Percentage of methods have been delivered*	In-person	70.8 (23.1)	75.0 [0, 100]
	Over the phone	14.8 (17.8)	10.0 [0, 90.0]
	Using video over the internet	14.5 (17.7)	10.0 [0, 75.0]

*Values are mean (SD) and Median [Min, Max].

### 3.2 Changes of physiotherapy practice in adapting telerehabilitation

Table 2 presents the previous and current experience with telerehabilitation of different rehabilitation activities. Therapists increased their delivery via telerehabilitation for all activities by several fold. For example, the use of a phone for assessments increased from 10.7% to 61.3% of PTs while videoconference increased from 5.3% to 70.7%. For exercise interventions, the use of a phone increased from 2.7% to 52.0%, while videoconference increased from 4.0% to 76.0%. The greatest use of telerehabilitation was with patient education (89.3% of PTs using phone and 80.0% using videoconference during the pandemic). The lowest use of telerehabilitation was with home environment assessment (37.3% of PTs using phone and 50.7% using videoconference). Home environment assessment was one of the few areas where videoconference was used by a higher proportion of PTs than by phone. PTs delivered a wide range of therapies by telerehabilitation; in addition to assessments and exercise, PTs managed to deliver pain management treatments, as well as address equipment needs by telerehabilitation.

**Table 2:** Previous and current experience with telerehabilitation (*n* = 75).

Categories		Experience prior to the pandemic	Currently delivering telerehabilitation during pandemic		
		Yes	Yes	No	N/A
Assessments	Phone	8 (10.7%)	46 (61.3%)	23 (30.7%)	6 (8.0%)
	Videoconference	4 (5.3%)	53 (70.7%)	12 (16.0%)	10 (13.3%)
Active exercise interventions	Phone	2 (2.7%)	39 (52.0%)	29 (38.7%)	7 (9.3%)
	Videoconference	3 (4.0%)	57 (76.0%)	7 (9.3%)	11 (14.7%)
Activity recommendations	Phone	11 (14.7%)	61 (81.3%)	9 (12.0%)	5 (6.7%)
	Videoconference	4 (5.3%)	58 (77.3%)	6 (8.0%)	11 (14.7%)
General follow-up	Phone	18 (24.0%)	64 (85.3%)	4 (5.3%)	7 (9.3%)
	Videoconference	4 (5.3%)	55 (73.3%)	8 (10.7%)	12 (16.0%)
Home environment safety/accessibility assessments	Phone	3 (4.0%)	28 (37.3%)	31 (41.3%)	16 (21.3%)
	Videoconference	1 (1.3%)	38 (50.7%)	17 (22.7%)	20 (26.7%)
Addressing equipment needs	Phone	12 (16.0%)	48 (64.0%)	19 (25.3%)	8 (10.7%)
	Videoconference	1 (1.3%)	47 (62.7%)	15 (20.0%)	13 (17.3%)
Pain management	Phone	11 (14.7%)	58 (77.3%)	9 (12.0%)	8 (10.7%)
	Videoconference	2 (2.7%)	51 (68.0%)	11 (14.7%)	13 (17.3%)
Patient-education	Phone	15 (20.0%)	67 (89.3%)	4 (5.3%)	4 (5.3%)
	Videoconference	4 (5.3%)	60 (80.0%)	5 (6.7%)	10 (13.3%)

Values are number (percent). The survey used a 5-point Likert scale and categories of “Strongly agreed” and “Agree” were consolidated into a “Yes” category, and “Strongly Disagree” and “Disagree” were consolidated into a “No” category.

### 3.3 Effectiveness of telerehabilitation

Table 3 presents the perceptions of PTs on whether they could effectively provide telerehabilitation over phone or videoconference. Overall, the PTs found videoconference to be a more effective delivery method than phone. For example, 28.0% felt that they could effectively initiate exercise prescription via phone, while 70.7% could by videoconference. However, less than half felt that they could use videoconference to effectively treat pain (49.3%), upper extremity function (40.0%) or strength and range of motion (48.0%). PTs did not think they could effectively deliver treatments that had a safety component; only 2.7% felt they could treat walking balance or mobility by phone, and 29.3% by videoconference. Importantly, 69.3% felt that they could effectively develop a therapeutic relationship with their clients by videoconference.

**Table 3:** Physiotherapists’ perception of effectively providing telerehabilitation over the phone or videoconference (*n* = 75).

Considering the majority of your client's needs		Agree	Undecided	Disagree	N/A
I can effectively perform a subjective assessment	Phone	57 (76.0%)	10 (13.3%)	4 (5.3%)	4 (5.3%)
	Videoconference	63 (84.0%)	3 (4.0%)	2 (2.7%)	7 (9.3%)
I can effectively perform an objective assessment	Phone	4 (5.3%)	17 (22.7%)	50 (66.7%)	4 (5.3%)
	Videoconference	29 (38.7%)	26 (34.7%)	13 (17.3%)	7 (9.3%)
I can effectively treat pain	Phone	17 (22.7%)	36 (48.0%)	16 (21.3%)	6 (8.0%)
	Videoconference	37 (49.3%)	21 (28.0%)	8 (10.7%)	9 (12.0%)
I can effectively treat walking balance and mobility	Phone	2 (2.7%)	16 (21.3%)	49 (65.3%)	8 (10.7%)
	Videoconference	22 (29.3%)	24 (32.0%)	19 (25.3%)	10 (13.3%)
I can effectively treat upper extremity function	Phone	5 (6.7%)	22 (29.3%)	40 (53.3%)	8 (10.7%)
	Videoconference	30 (40.0%)	21 (28.0%)	13 (17.3%)	11 (14.7%)
I can effectively treat strength and range of motion	Phone	11 (14.7%)	16 (21.3%)	41 (54.7%)	7 (9.3%)
	Videoconference	36 (48.0%)	18 (24.0%)	11 (14.7%)	10 (13.3%)
I can effectively communicate exercise instructions	Phone	27 (36.0%)	23 (30.7%)	20 (26.7%)	5 (6.7%)
	Videoconference	56 (74.7%)	8 (10.7%)	3 (4.0%)	8 (10.7%)
I can effectively initiate exercise prescriptions	Phone	21 (28.0%)	15 (20.0%)	33 (44.0%)	6 (8.0%)
	Videoconference	53 (70.7%)	4 (5.3%)	9 (12.0%)	9 (12.0%)
I can effectively progress the intensity of exercise prescriptions	Phone	36 (48.0%)	17 (22.7%)	14 (18.7%)	8 (10.7%)
	Videoconference	51 (68.0%)	10 (13.3%)	3 (4.0%)	11 (14.7%)
I can effectively develop a therapeutic relationship with my clients	Phone	37 (49.3%)	24 (32.0%)	10 (13.3%)	4 (5.3%)
	Videoconference	52 (69.3%)	11 (14.7%)	4 (5.3%)	8 (10.7%)
I can effectively prescribe equipment	Phone	17 (22.7%)	18 (24.0%)	31 (41.3%)	9 (12.0%)
	Videoconference	35 (46.7%)	14 (18.7%)	14 (18.7%)	12 (16.0%)

Values are number (percent).

### 3.4 Barriers and facilitators in implementing telerehabilitation services

Table 4 presents the perceived barriers by the PTs when offering telerehabilitation. Whether by phone or videoconference, over half of the PTs identified physical safety of their clients and difficulty for clients with hearing loss as barriers. In addition, the most common technological barriers over videoconference that the respondents experienced were client comfort with equipment (90.7%) and positioning of the webcam (76.0%). Other technological barriers, such as internet stability (68.0%) and sound problems (61.3%) were also common.

**Table 4:** Barriers to providing telerehabilitation (*n* = 75).

What barriers have you experienced in order to offer telerehabilitation over___?	Phone	Internet video
Client interest/engagement	32 (42.7%)	40 (53.3%)
Physical safety of clients	41 (54.7%)	40 (53.3%)
Difficulty for clients with hearing loss	44 (58.7%)	41 (54.7%)
Difficulty for clients with speech impairment	24 (32.0%)	16 (21.3%)
Difficulty for clients with cognitive impairment	37 (49.3%)	36 (48.0%)
What technological barriers have you experienced when offering telerehabilitation using video over the internet?		
Personal comfort	n/a	32 (42.7%)
Client comfort		68 (90.7%)
Internet stability		51 (68.0%)
Positioning of webcam		57 (76.0%)
Clarity of video		38 (50.7%)
Sound problems		46 (61.3%)

Values are number (percent).

In Table 5, barriers and facilitators were explored in more detail. When providing telerehabilitation over telephone or videoconference, the majority of PTs agreed that they had the necessary space (68.0% and 61.3%, respectively) or equipment (73.3% and 80.0%, respectively), and they were also able to maintain client privacy (74.7% and 65.3%, respectively). Additionally, for providing videoconference, the majority of PTs agreed that it can effectively engage caregivers as a partner in the rehabilitation in the home (61.3%), and PTs also felt sufficiently trained to use the technology (70.7%) and to optimize communication with clients (54.7%).

**Table 5:** Physiotherapists’ perceived barriers or facilitators to implementation telerehabilitation services (*n* = 75).

Statement		Agree	Undecided	Disagree	N/A
When providing telerehabilitation over the ___, I have space needed. (e.g., confidential, quiet)	Phone	51 (68.0%)	11 (14.7%)	13 (17.3%)	\
	Videoconference	46 (61.3%)	14 (18.7%)	15 (20.0%)	\
When providing telerehabilitation over the ___, I have all the equipment I need. (e.g., an appropriate phone/computer)	Phone	55 (73.3%)	9 (12.0%)	11 (14.7%)	\
	Videoconference	60 (80.0%)	9 (12.0%)	6 (8.0%)	\
My assessments are as effective over the ___ as face to face assessments	Phone	5 (6.7%)	14 (18.7%)	56 (74.7%)	\
	Videoconference	15 (20.0%)	24 (32.0%)	36 (48.0%)	\
When treating clients over the ___, concerns for client safety limits my treatment program I implement	Phone	52 (69.3%)	16 (21.3%)	7 (9.3%)	\
	Videoconference	46 (61.3%)	20 (26.7%)	9 (12.0%)	\
The inability to incorporate hands-on techniques (e.g., applied to soft-tissue, manual therapy, TENS, FES) while providing telerehabilitation over the ___ negatively impacts client outcomes.	Phone	37 (49.3%)	16 (21.3%)	10 (13.3%)	12 (16.0%)
	Videoconference	36 (48.0%)	19 (25.3%)	11 (14.7%)	9 (12.0%)
Inability to provide standby assistance or contact assistance during gait and mobility training (including transfer training), while providing telerehabilitation over the ___, negatively impacts client outcomes.	Phone	57 (76.0%)	6 (8.0%)	2 (2.7%)	10 (13.3%)
	Videoconference	51 (68.0%)	10 (13.3%)	7 (9.3%)	7 (9.3%)
I can offer telerehabilitation over the ___ and maintain client privacy	Phone	56 (74.7%)	15 (20.0%)	4 (5.3%)	\
	Videoconference	49 (65.3%)	23 (30.7%)	3 (4.0%)	\
Telerehabilitation over the ___ resulted in clients achieving confidence in their abilities equivalent to face to face services. (i.e., confidence in function in a day to day capacity, managing their condition, their ability to move).	Phone	11 (14.7%)	32 (42.7%)	32 (42.7%)	\
	Videoconference	25 (33.3%)	26 (34.7%)	24 (32.0%)	\
Telerehabilitation over the ___ effectively engages caregivers (or family members, social supports) as a partner in rehabilitation in the home.	Phone	28 (37.3%)	24 (32.0%)	23 (30.7%)	\
	Videoconference	46 (61.3%)	23 (30.7%)	6 (8.0%)	\
Telerehabilitation over the ___ places too much demand on caregivers (or family members, social supports) in the home to support the call/tech	Phone	22 (29.3%)	29 (38.7%)	24 (32.0%)	\
	Videoconference	28 (37.3%)	26 (34.7%)	21 (28.0%)	\
Telerehabilitation over the ___ places too much demand on caregivers (or family members, social supports) in the home to physically support rehab efforts	Phone	26 (34.7%)	36 (48.0%)	13 (17.3%)	\
	Videoconference	23 (30.7%)	33 (44.0%)	19 (25.3%)	\
Telerehabilitation over the ___ is limited when the client lives alone	Phone	36 (48.0%)	20 (26.7%)	19 (25.3%)	\
	Videoconference	36 (48.0%)	19 (25.3%)	20 (26.7%)	\
Delivering telerehabilitation over the ___ is tiring for me as a physiotherapist	Phone	32 (42.7%)	21 (28.0%)	22 (29.3%)	\
	Videoconference	39 (52.0%)	12 (16.0%)	24 (32.0%)	\
I feel sufficiently trained to use the technology needed to use video over the internet to provide telerehabilitation for clients.	Videoconference	53 (70.7%)	11 (14.7%)	11 (14.7%)	\
I feel sufficiently trained to optimize communication with clients while using video over the internet to provide telerehabilitation	Videoconference	41 (54.7%)	27 (36.0%)	7 (9.3%)	\
Considering the typical rehabilitation needs of my clients, I am able to address the goals of my clients using video over the internet as well as during face to face treatment.	Videoconference	25 (33.3%)	23 (30.7%)	27 (36.0%)	\
All of my clients on my caseload have access to technology required to receive telerehabilitation using video over the internet	Videoconference	9 (12.0%)	8 (10.7%)	58 (77.3%)	\
I can cover more clinical content in a single session using video over the internet compared to face to face	Videoconference	4 (5.3%)	20 (26.7%)	51 (68.0%)	\
Following COVID 19 pandemic, I will continue to integrate telerehabilitation over the ___ into my practice	Phone	41 (54.7%)	16 (21.3%)	18 (24.0%)	\
	Videoconference	51 (68.0%)	13 (17.3%)	11 (14.7%)	\
Following COVID 19 pandemic, clients will continue to request telerehabilitation services over the ___ to address their rehabilitation needs	Phone	24 (32.0%)	27 (36.0%)	24 (32.0%)	\
	Videoconference	32 (42.7%)	30 (40.0%)	13 (17.3%)	\

Values are number (percent).

However, regardless of delivering telerehabilitation over phone or videoconference, client safety was a perceived barrier that limited their treatment program (69.3% and 61.3%, respectively). Additionally, the majority of respondents agreed that the inability to provide standby assistance during gait and mobility training negatively impacted client outcomes (76.0% and 68.0%, respectively). Specific to delivering telerehabilitation over phone, the majority of PTs disagreed that performing assessments over phone were as effective as an in-person assessment (74.7%). Specific to delivering telerehabilitation over videoconference, PTs agreed that it was tiring for them (52.0%) and disagreed that they can cover more clinical content in a single session compared to face to face (68.0%). In addition, the majority of PTs responded that not all clients on their caseloads had access to technology (77.3%). Nevertheless, following the COVID 19 pandemic, more than half of the PTs agreed that they would continue to integrate telerehabilitation over phone or videoconference into their practice (54.7% and 68.0%, respectively).

## 4 Discussion

We captured the responses of approximately half of the PTs who provided outpatients services in a large public health authority in Canada. The respondents are representative of the American PT sector with mainly women who are typically 30–50 years of age, with most with less than 10 years of practice (10). Although the majority of this health authority is situated in urban cities (Vancouver and Richmond, British Columbia), the authority also serves several rural communities. These data were collected approximately one year after the pandemic started, so most PTs would have had several months of experience utilizing telerehabilitation by this time.

Our results concur with others who have reported on the substantial increase in the use of telerehabilitation with PTs over the pandemic (2, 6, 11). Only a few of our respondents had used videoconference for rehabilitation prior to the pandemic. While our findings agree with others that have noted safety concerns with telerehabilitation (11), our study further identified the specific activities that therapists found challenging or ineffective for use over a telerehabilitation platform. The majority of respondents found videoconference effective for exercise prescription, including progressing the intensity of exercise. However, while they could prescribe exercise, less than half felt that they could effectively treat strength, range of motion or pain over videoconference, and less than one-third felt they could effectively treat walking or mobility over videoconference. Improving strength, range, pain and mobility are essential components of a PT's job. Ensuring a client's physical safety was identified as one of the barriers for improving walking and mobility using telerehabilitation. This reflects the requirement of standby or contact assistance that PTs provide during gait and mobility training across many patient groups.

Telerehabilitation has been suggested to be a potential mode of healthcare delivery to address accessibility to health care services in Canada. However, the majority of PTs reported that not all of the clients on their caseload had access to the technology required to receive telerehabilitation using video over the internet. Furthermore, almost half of all PTs agreed the use of telerehabilitation services was limited when clients lived alone. Contextually, older adults (age 65+) are an important sector of the population that access physiotherapy services. In Canada, almost one-third (27.9%) of older adults living in independent dwellings reported living alone (12). Taken together, concerns regarding access to technology and living alone present challenges to the potential reach and ability of telerehabilitation to equitably address underserved clinical groups and regions of the province (e.g., rural).

Interestingly, when caregivers were present, almost all PTs (over 90%) agreed that telerehab over videoconference effectively engages caregivers (or family members, social supports) as a partner in rehabilitation in the home. However, it is important to consider that PTs also noted the potential additional burden that this engagement places on caregivers. Therefore, it is recommended that this be a consideration of the development of any future telerehabilitation programs or services and that PTs check-in with caregivers regarding potential additional burden experienced. Further challenging the effective use of telerehabilitation, almost half of all PTs felt the use of telerehabilitation services was limited when clients lived alone.

It is concerning that almost half of the PTs that participated in this survey reported telerehabilitation to be tiring. It is important to note that this survey took place during the pandemic, a time which placed considerable additional burden on health care providers. However, telerehabilitation may also be tiring as a result of the high percentage of barriers reported that needed to be addressed (e.g., technological challenges, client interest, safety). Additionally, PTs overwhelmingly felt that they could not cover as much clinical content in a single session using videoconference compared to face to face. Finally, telerehabilitation commonly requires increased communication to set-up the session, and develop instructional materials to send the client in addition to teaching during the session.

### 4.1 Limitations

It is possible that PTs who responded to a survey about telerehabilitation were more likely to have tried telerehabilitation or have had positive experiences. These individuals may be more willing to share their experiences and more likely to respond than those who did not have positive experiences. When we interpreted our results, we were aware that both the sample size and the nonresponse rate found in this survey are limitations. While we used reminders and stipends to increase our response rate, the response rate may have been impacted by COVID-19, with health care providers experiencing burn-out, fatigue as well as illness. While we do not have information about the non-responders to our survey, we had a very targeted population of a large health authority that matched the typical public health sector PT demographic.

## 5 Conclusion

Despite all the barriers that the PTs reported, they still felt positive to continue integrating telerehabilitation into clinical practicing following the pandemic. This finding was in line with previous studies that majority of the PTs had hold positive attitudes towards telerehabilitation services and their integration to clinical practices (6, 13). Specifically, our findings suggest that telerehabilitation may be used for follow-up and potentially for uncomplicated progression of activity or exercise programs.

## Data Availability

The original contributions presented in the study are included in the article/Supplementary Material, further inquiries can be directed to the corresponding author.
