# Supplementary material for: A survey of the experiences of delivering physiotherapy services through telerehabilitation during the COVID-19 pandemic
Source: Front Rehabil Sci. 2024 Oct 24;5:1486801. doi: 10.3389/fresc.2024.1486801 (PMC11540677; doi:10.3389/fresc.2024.1486801)
Supplement: Supplementary file 1 [file Datasheet1.pdf]

Title: **Barriers and Facilitators to virtual physical therapy due to the COVID-19 pandemic**

Thank you for taking the time to let us know about your experience with tele-rehabilitation.

Tele-rehabilitation: "The delivery of Physiotherapy via information and communication technologies such as phone or use of video over the internet (e.g. Zoom, Face Time)." For the purposes of this survey, Tele-rehabilitation (or Tele-rehab) includes all aspects of physiotherapy intervention, such as assessment, education, and treatment services provided to clients.

This study is run by a team made-up of researchers, clinicians and VCH leadership currently collaborating in the Vancouver Coastal Health Research Institute (VCHRI) Rehabilitation Research Program.

To participate in this study, you must be at least 18 years old and read and understand English. You also have to be a physiotherapist registered in the province of British Columbia, employed by Vancouver Coastal Health.

Survey will cover:

1) Information about you as a physiotherapist
2) Your current and previous experience with tele-rehabilitation
3) Your views regarding your use of tele-rehabilitation during the current pandemic and beyond with each mode of delivery:
    a. Tele-rehab over the phone
    b. Tele-rehab using video over the Internet

Your responses will help us to better understand clinicians' experiences with Tele-rehabilitation and will inform VCH's approach to tele-rehabilitation. The survey will take about 30 minutes to complete and your answers will be anonymous. You will be assigned a unique study number as a participant in this study.

You will be compensated with $50 for completing the survey. At the end of the study, you will be asked to provide your VCH email or mailing address to receive your reimbursement and to be contacted for further research opportunity if you indicate you are interested. This information will be used to verify your employment at VCH.

Most importantly we want to hear from as many physiotherapists voices as possible. Some questions have set responses and others will include open-ended questions where you can tell us your thoughts more directly and comprehensively. If we are not addressing important aspects – tell us – we really want to hear what you think.

*****It is recommended that you use a computer or tablet to answer this survey. Smartphone screens will be too small to read or answer questions. Google Chrome is recommended. Please complete the survey in one sitting, partial completion of the survey will not be saved. *****

If you start the survey and decide you no longer wish to participate: you can close the page without giving any specific reasons. Your incomplete responses will be deleted and will not be analyzed. However, you will only be reimbursed if you complete the survey.

Please note that quotes from the open-ended questions may be used in published reports. However, the quotes used will not be accompanied by any descriptive information that would identify you. Therefore, no additional risk for participants will result from using quotes to capture common themes of the findings of the survey. Once reports are submitted for publication, participants will not be able to withdraw their data.

Should you have any questions about this study, feel free to contact:
Dr. Courtney Pollock (PT, PhD; Principal Investigator) at courtney.pollock@ubc.ca

If you have any concerns or complaints about your rights as a research participant and/or your experiences while participating in this study, contact the Research Participant Complaint Line in the University of British Columbia Office of Research Ethics by e-mail at RSIL@ors.ubc.ca or by phone at 604-822-8598 (Toll Free: 1-877-822-8598). Please reference the study number [H20-01550] when calling so the Complaint Line staff can better assist you.

Thank you from the Study Team:

Dr. Courtney Pollock (PT, PhD, Principal Investigator)
Dr. Janice Eng (OT/PT, PhD, Coinvestigator)
Ms. Catherine Le Cornu Levett (PT MSc, Coinvestigator)
Ms. Anne Harris (MSc PT, Coinvestigator)
Dr. Jennifer Yao (MD, Coinvestigator)
Dr. Amy Schneeberg (PT, PhD, Coinvestigator)
Tzu-Hsuan Peng (OT, MSc student)

Click the NEXT button if you consent to participate.

PLEASE ANSWER THESE QUESTIONS IN RELATION TO THE CURRENT STATE OF PRACTICE

1. **Where do you spend the majority of your work time?**
   - ○ Acute care
   - ○ Outpatient
   - ○ Community/home care
   - ○ Inpatient rehabilitation

2. Please specify your program (e.g. inpatient adult neurology, outpatient adult MSK, telehealth innovation deployment education and sustainment (TIDES), intensive rehab day program (IRDP)):

   [ ]

3. **Your current physiotherapy field for the majority of your work time:**
   - ○ Adults

○ Pediatrics

4. **Your current physiotherapy practice for the majority of your work time:**

○ Arthritis care
○ Mental health
○ Neuro
○ Spine
○ Orthopaedics
○ Hand therapy
○ Mixed/general caseload
○ Cardio-respiratory and pulmonary rehab
Other: Please specify ______________________

YOUR PREVIOUS EXPERIENCE WITH TELE-REHABILITATION

1. **Prior to COVID-19, how many clients were you seeing face to face per day on average?**

   [ ] /per day

2. **Prior to COVID-19, had you ever delivered tele-rehab for clients over the phone?**
   - ○ No
   - ○ Yes

3. **Please tick all boxes that describe tele-rehab you provided over the phone:**

☐ Assessments
☐ Active exercise interventions (e.g. teaching of exercises, movement retraining)
☐ Activity recommendations (e.g. prescribing and progressing home exercise/walking programs)
☐ General follow-up (e.g. discussing current status and any concerns)
☐ Home environment safety/accessibility assessments
☐ Addressing equipment needs
☐ Pain management
☐ Patient-education
☐ Other please specify:_____________

4. **Prior to COVID-19, had you ever delivered tele-rehab for clients via video over the internet?**
   - ○ No
   - ○ Yes

5. **Please tick all boxes that describe tele-rehab you provided via video over the internet:**

☐ Assessments
☐ Active exercise interventions (e.g. teaching of exercises, movement retraining)
☐ Activity recommendations (e.g. prescribing and progressing home exercise/walking programs)
☐ General follow-up (e.g. discussing current status and any concerns)
☐ Home environment safety/accessibility assessments

☐ Addressing equipment needs
☐ Pain management
☐ Patient-education
☐ Other please specify:_____________

YOUR CURRENT PRACTICE AND EXPERIENCE WITH TELE-REHABILITATION

1. **Do you currently offer physiotherapy face to face for any client group?**
   ◯ No
   ◯ Yes

2. **On average, over the past 2 weeks, how many clients per day did you provide physiotherapy face to face?**
   ☐ /per day

3. **Do you currently offer tele-rehab over the phone for any client group?**
   ◯ *No, it is not working for me. Please describe why it is not working:*
   ☐
   ◯ *Yes. Please specify the client groups you are serving:*
   ☐
   ◯ *Yes, in a limited fashion. Please list client groups AND challenges/barriers that make this a limited option:*
   ☐

4. **On average, over the past 2 weeks, how many clients per day did you provide tele-rehab for over the phone?**
   ☐ /per day

5. **Considering your typical practice day, to the best of your ability, please rate the following practice activities you currently deliver using the phone:**

	**Often** deliver over the phone	**Sometimes** deliver over the phone	**Seldom** deliver over the phone	**Never** deliver over the phone	**N/A -** I do not do this in my practice
Assessments	O	O	O	O	O
Active exercise interventions (e.g. teaching of exercises, movement retraining)	O	O	O	O	O
Activity recommendations (e.g. prescribing and	O	O	O	O	O

progressing home exercise/walking programs)					
General follow-up (e.g. discussing current status and any concerns)	O	O	O	O	O
Home environment safety/accessibility assessments	O	O	O	O	O
Addressing equipment needs	O	O	O	O	O
Pain management	O	O	O	O	O
Patient-education	O	O	O	O	O
Other please specify:_____________	O	O	O	O	O

6. **Do you currently offer tele-rehab using video over the internet for any client group?**

○ *No, it is not working for me. Please describe why it is not working:*

[ ]

○ *Yes. Please specify the client groups you are serving:*

[ ]

○ *Yes, in a limited fashion. Please list client groups AND challenges/barriers that make this a limited option:*

[ ]

7. **On average, over the past 2 weeks, how many clients per day did you provide tele-rehab via video over the internet**

[ ] /per day

8. **Considering your typical practice day, to the best of your ability, please rate the following practice activities you currently deleiver using video over the internet:**

	**Often** deliver via video over the internet	**Sometimes** deliver via video over the internet	**Seldom** deliver via video over the internet	**Never** deliver via video over the internet	**N/A -** I do not do this in my practice
Assessments	O	O	O	O	O
Active exercise interventions (e.g. teaching of exercises, movement retraining)	O	O	O	O	O
Activity recommendations (e.g. prescribing and progressing home exercise/walking programs)	O	O	O	O	O

General follow-up (e.g. discussing current status and any concerns)	O	O	O	O	O
Home environment safety/accessibility assessments	O	O	O	O	O
Addressing equipment needs	O	O	O	O	O
Pain management	O	O	O	O	O
Patient-education	O	O	O	O	O
Other please specify:_____________	O	O	O	O	O

9. **How many minutes does a typical session using video over internet require compared to a face-to-face session (including preparation/set-up time, intervention, charting time, etc.).**
   ______ minutes for one client using video over internet
   ______ minutes for one client face-to-face session

10. **What percentage of each of the following methods have you delivered physiotherapy over the past 2 weeks? (Total must sum to 100)**
    Fact-to-face:_______
    Over the phone:________
    Via video over the internet:_______

Notes: IF PARTICIPANTS HAVE NOT PARTICIPATED IN EITHER TELEPHONE OR VIDEO SKIP TO NEXT SECTION FUNCTION

Use of **phone** as the platform for tele-rehab

The following are questions related to your experience delivering tele-rehab over the phone. This section includes at least 17 questions. Please keep in mind that you may not be able to revise your answers once you finish the whole section.

1. **What barriers have you experienced in order to offer tele-rehab over the phone**? (Please select all that apply)

- ○ Client interest/engagement
- ○ Physical safety of clients
- ○ Difficulty for clients with hearing loss
- ○ Difficulty for clients with speech impairment
- ○ Difficulty for clients with cognitive impairment
- ○ Other, please specify:

2. The majority of your clients have reported tele-rehab over the phone to be:

- ○ Better than in person
- ○ Equally good to in person
- ○ Not as helpful as in person, but better than nothing
- ○ Not useful
- ○ No feedback received
- ○ N/A – Not applicable

Please provide details:

3. Did you have to make more supplemental material to provide to clients when offering tele-rehab over the phone compared to face to face?

- ○ No
- ○ Yes, please describe:

4. **When providing tele-rehab over the phone, I have space needed. (e.g. confidential, quiet)**

Strongly agree *Agree* *Undecided* *Disagree* *Strongly disagree*

If you do not what do you need___________________

5. When providing tele-rehab over the phone, I have all the equipment I need. (e.g. an appropriate phone)

Strongly agree *Agree* *Undecided* *Disagree* *Strongly disagree*

If not, what additional equipment do you need to provide tele-rehab over the phone_____________

What additional accessories assist you to provide tele-rehab over the phone____________

6. Considering the majority of your client's needs, providing tele-rehab over the phone:
I can effectively perform a subjective assessment.

Strongly agree *Agree* *Undecided* *Disagree* *Strongly disagree* *N/A – I do not do this in my practice*

I can effectively perform an objective assessment

Strongly agree *Agree* *Undecided* *Disagree* *Strongly disagree* *N/A – I do not do this in my practice*

I can effectively treat pain.

Strongly agree Agree Undecided Disagree Strongly disagree N/A – I do not do this in my practice

I can effectively treat walking balance and mobility.

Strongly agree Agree Undecided Disagree Strongly disagree N/A – I do not do this in my practice

I can effectively treat upper extremity function.

Strongly agree Agree Undecided Disagree Strongly disagree N/A – I do not do this in my practice

I can effectively treat strength and range of motion.

Strongly agree Agree Undecided Disagree Strongly disagree N/A – I do not do this in my practice

I can effectively communicate exercise instructions.

Strongly agree Agree Undecided Disagree Strongly disagree N/A – I do not do this in my practice

I can effectively initiate exercise prescriptions.

Strongly agree Agree Undecided Disagree Strongly disagree N/A – I do not do this in my practice

I can effectively progress the intensity of exercise prescriptions.

Strongly agree Agree Undecided Disagree Strongly disagree N/A – I do not do this in my practice

I can effectively develop a therapeutic relationship with my clients.

Strongly agree Agree Undecided Disagree Strongly disagree N/A – I do not do this in my practice

I can effectively prescribe equipment.

Strongly agree Agree Undecided Disagree Strongly disagree N/A – I do not do this in my practice

Please specify the equipment you typically prescribe:

7. **My assessments are as effective over the phone as face to face assessments.**

Strongly agree Agree Undecided Disagree Strongly disagree

Which outcome measures have you been able to use over the phone: ____________________________

8. **When treating clients over the phone, concerns for client safety limits my treatment program I implement.**

Strongly agree Agree Undecided Disagree Strongly disagree

Describe your concerns for safety and/or any incidents that have occurred: _________________

9. **The inability to incorporate hands-on techniques (e.g. applied to soft-tissue, manual therapy, TENS, FES) while providing tele-rehab over the phone negatively impacts client outcomes.**

Strongly agree   Agree   Undecided   Disagree   Strongly disagree   N/A – I do not do this in my practice

10. **Inability to provide standby assistance or contact assistance during gait and mobility training (including transfer training), while providing tele-rehab over the phone, negatively impacts client outcomes.**

Strongly agree   Agree   Undecided   Disagree   Strongly disagree   N/A – I do not do this in my practice

11. **I can offer tele-rehab over the phone and maintain client privacy.**

Strongly agree   Agree   Undecided   Disagree   Strongly disagree

Tell us more about your choice: __________________

12. **Tele-rehab over the phone resulted in clients achieving confidence in their abilities equivalent to face to face services. (i.e., confidence in function in a day to day capacity, managing their condition, their ability to move).**

Strongly agree   Agree   Undecided   Disagree   Strongly disagree

13. **Tele-rehab over the phone effectively engages caregivers (or family members, social supports) as a partner in rehabilitation in the home.**

Strongly agree   Agree   Undecided   Disagree   Strongly disagree

14. **Tele-rehab over the phone places too much demand on caregivers (or family members, social supports) in the home,**

To support the call:

Strongly agree   Agree   Undecided   Disagree   Strongly disagree

To physically support rehab efforts:

Strongly agree   Agree   Undecided   Disagree   Strongly disagree

15. **Tele-rehab over the phone is limited when the client lives alone.**

Strongly agree *Agree* *Undecided* *Disagree* *Strongly disagree*

16. **Delivering tele-rehab over the phone is tiring for me as a physiotherapist.**

Strongly agree *Agree* *Undecided* *Disagree* *Strongly disagree*

*If agree, please explain why:*____

17. **What are your top 3 recommendations for physiotherapists new to providing tele-rehab over the phone**?

Tele-rehabilitation via video over the internet (e.g. Zoom, Face Time, Skype)

The following are questions related to your experience delivering tele-rehab using video over the internet. This section includes at least 28 questions. Please keep in mind that you may not be able to revise your answers once you finish the whole section.

1. **Which video chat services do you use? (Please select all that apply)**
   - ○ Zoom
   - ○ Face Time
   - ○ Skype
   - ○ Microsoft Team
   - ○ Others: ________

2. **Please rate the following devices that you use when offering tele-rehab using video over the internet.**

	Often use	Sometimes use	Seldom use	Never use
Mobile phone	O	O	O	O
Tablet	O	O	O	O
Laptop computer	O	O	O	O
Desk-top computer	O	O	O	O

3. **Please rate the following devices that your clients use when receiving tele-rehab using video over the internet.**

	Often use	Sometimes use	Seldom use	Never use
Mobile phone	O	O	O	O
Tablet	O	O	O	O
Laptop computer	O	O	O	O

Desk-top computer	O	O	O	O

4. What technological barriers have you experienced when offering tele-rehab using video over the internet? (Please select all that apply)?

- ○ Personal comfort/knowledge with use of required technology
- ○ Client comfort/knowledge with use of required technology
- ○ Internet stability
- ○ Positioning of webcam
- ○ Clarity of video
- ○ Sound problems
- ○ Other, please specify:

5. Aside from technology, what other barriers have you experienced in order to offer tele-rehab using video over the internet?

- ○ Client interest/engagement
- ○ Physical safety of clients
- ○ Difficulty of platform for clients with hearing loss
- ○ Difficulty of platform for clients with speech impairment
- ○ Difficulty of platform for clients with cognitive impairment
- ○ Other, please specify:

6. **The majority of your clients have reported tele-rehab using video over the internet to be**: (Please select all that apply)

- ○ Better than in person
- ○ Equally good to in person
- ○ Not as helpful as in person, but better than nothing
- ○ Not useful
- ○ No feedback received
- ○ N/A – Not applicable

Please provide details:

7. I feel sufficiently trained to use the <u>technology needed</u> to use video over the internet to provide tele-rehab for clients.

Strongly agree *Agree* *Undecided* *Disagree* *Strongly disagree*

Specify training informal or formal (e.g. workshops, resources, information from co-worker):

____

8. I feel sufficiently trained to optimize <u>communication</u> with clients while using video over the internet to provide tele-rehab.

Strongly agree   Agree   Undecided   Disagree   Strongly disagree

Specify training informal or formal (e.g. workshops, resources, information from co-worker):

____

9. **I have sufficient physical space to provide tele-rehab using video over the internet. (e.g. confidential, quiet)**

Strongly agree   Agree   Undecided   Disagree   Strongly disagree

If you do not, what do you need? ___________________

10. I have sufficient equipment to provide tele-rehab using video over the internet. (e.g. an appropriate computer)

Strongly agree   Agree   Undecided   Disagree   Strongly disagree

If not, what additional equipment do you need to provide tele-rehab using video over the internet? _____________

What additional accessories assist you to provide tele-rehab using video over the internet? ____________

11. Did you have to make more supplemental material to provide to clients when providing tele-rehab using video over the internet compared to face to face?

- ○ No
- ○ Yes, please describe:

12. Considering the majority of your client's needs, providing tele-rehab via video over the internet:

I can effectively perform a subjective assessment.

Strongly agree   Agree   Undecided   Disagree   Strongly disagree   N/A – I do not do this in my practice

I can effectively perform an objective assessment.

Strongly agree Agree Undecided Disagree Strongly disagree N/A – I do not do this in my practice

I can effectively treat pain.

Strongly agree Agree Undecided Disagree Strongly disagree N/A – I do not do this in my practice

I can effectively treat walking balance and mobility.

Strongly agree Agree Undecided Disagree Strongly disagree N/A – I do not do this in my practice

I can effectively treat upper extremity function.

Strongly agree Agree Undecided Disagree Strongly disagree N/A – I do not do this in my practice

I can effectively treat strength and range of motion.

Strongly agree Agree Undecided Disagree Strongly disagree N/A – I do not do this in my practice

I can effectively communicate exercise instructions.

Strongly agree Agree Undecided Disagree Strongly disagree N/A – I do not do this in my practice

I can effectively initiate exercise prescriptions.

Strongly agree Agree Undecided Disagree Strongly disagree N/A – I do not do this in my practice

I can effectively progress the intensity of exercise prescriptions.

Strongly agree Agree Undecided Disagree Strongly disagree N/A – I do not do this in my practice

I can effectively develop a therapeutic relationship with my clients.

Strongly agree Agree Undecided Disagree Strongly disagree N/A – I do not do this in my practice

I can effectively prescribe equipment.

Strongly agree Agree Undecided Disagree Strongly disagree N/A – I do not do this in my practice

Please specify the equipment you typically prescribe:

13. **My assessments are as effective using video over the internet compared to face to face assessments.**

Strongly agree Agree Undecided Disagree Strongly disagree

Which outcome measures have you ***been able to*** *use via video over the internet:* ___________________

Which outcome measures have you ***been unable to*** *use via video over the internet:* _________________

14. Please describe any areas of physiotherapy treatment that you have been unable to provide using video over the internet:

15. **Considering the typical rehabilitation needs of my clients, I am able to address the goals of my clients using video over the internet as well as during face to face treatment.**

Strongly agree *Agree* *Undecided* *Disagree* *Strongly disagree*

16. All of my clients on my caseload have access to technology required to receive tele-rehab using video over the internet.

Strongly agree *Agree* *Undecided* *Disagree* *Strongly disagree*

*If not, please explain why*________________________________

17. When treating clients using video over the internet, concerns for client safety limit the treatment program I implement.

Strongly agree *Agree* *Undecided* *Disagree* *Strongly disagree*

Describe your concerns for safety and/or any incidents that have occurred: _________________

18. **I can offer tele-rehab using video over the internet and maintain client privacy.**

Strongly agree *Agree* *Undecided* *Disagree* *Strongly disagree*

Tell us more about your choice: __________________

19. I can cover more clinical content in a single session using video over the internet compared to face to face.

Strongly agree *Agree* *Undecided* *Disagree* *Strongly disagree*

20. **The inability to incorporate hands-on techniques (e.g. applied to soft-tissue, manual therapy) while providing tele-rehab using video over the internet negatively impact client outcomes.**

Strongly agree *Agree* *Undecided* *Disagree* *Strongly disagree* *N/A – I do not do this in my practice*

21. Inability to provide standby assistance or contact assistance during gait and mobility training (including transfer training), while providing tele-rehab using video over the internet, negatively impact client outcomes.

Strongly agree Agree Undecided Disagree Strongly disagree N/A – I do not do this in my practice

22. Tele-rehab using video over the internet resulted in clients achieving confidence in their abilities no differently than face to face services. (i.e., confidence in function in a day to day capacity, managing their condition, their ability to move).

Strongly agree Agree Undecided Disagree Strongly disagree

23. Tele-rehab using video over the internet effectively engages caregivers (or family members, social supports) as a partner in rehabilitation in the home.

Strongly agree Agree Undecided Disagree Strongly disagree

24. Physiotherapy services using video over the internet places too much demand on caregivers (or family members, social supports) in the home,

To support tech:

Strongly agree Agree Undecided Disagree Strongly disagree

To physically support rehab efforts:

Strongly agree Agree Undecided Disagree Strongly disagree

25. Tele-rehab using video over the internet is limited when the client lives alone.

Strongly agree Agree Undecided Disagree Strongly disagree

26. **Delivering tele-rehab using video over the internet is tiring for me as a physiotherapist.**

Strongly agree Agree Undecided Disagree Strongly disagree

*If agree, please explain why:*____

27. When providing tele-rehab via video over the internet, please specify any limitations in treatment due to the inability to access typical therapy equipment you normally use face to face? (e.g. parallel bars)

28. **What are your top 3 recommendations for physiotherapists new to providing tele-rehab using video over the internet**?

INFORMATION ABOUT YOU AS A PHYSIOTHERAPIST:

1. **Age range:**
   - ○ 21-30 years old
   - ○ 31-40 years
   - ○ 41-50 years
   - ○ 51-60 years
   - ○ 61-70 years

2. **Are you:**
   - ○ Male
   - ○ Female
   - ○ Non-binary
   - ○ Prefer not to answer

3. **How long have you been a practicing physiotherapist?**
   - ○ 0-5 years
   - ○ 6-10
   - ○ 11-15
   - ○ 16-20
   - ○ 21-25
   - ○ >25

4. **Do you have any postgraduate qualifications?**
   - ○ Yes – specify: ___________________________
   - ○ No

5. **Which is your Community of Care:**
   - ○ Richmond
   - ○ Vancouver
   - ○ Coastal (sea to sky and sunshine coast)
   - ○ North Shore

6. **Are you …**
   - ○ Full-Time
   - ○ Part-Time, please specify hours per week___________

7. **How long have you been in your current area of practice:**
   - ○ 0-5 years
   - ○ 6-10 years
   - ○ 11-15 years
   - ○ 16-20 years
   - ○ 21-25 years
   - ○ >25

8. **Following COVID 19 pandemic, I will continue to integrate tele-rehab over the phone into my practice.**

Strongly agree *Agree* *Undecided* *Disagree* *Strongly disagree*

9. **Following COVID 19 pandemic, I will continue to integrate tele-rehab using video over internet into my practice.**

Strongly agree *Agree* *Undecided* *Disagree* *Strongly disagree*

10. **Following COVID 19 pandemic, clients will continue to request tele-rehab services over the phone to address their rehabilitation needs.**

Strongly agree *Agree* *Undecided* *Disagree* *Strongly disagree*

11. **Following COVID 19 pandemic, clients will continue to request tele-rehab services using video over internet to address their rehabilitation needs.**

Strongly agree *Agree* *Undecided* *Disagree* *Strongly disagree*

CONTACT INFORMATION FOR REIMBURSEMENT AND/OR FUTURE CONTACT:

Thank you for completing this survey! We appreciate you taking the time to let us know your thoughts and opinions regarding tele-rehabilitation. You will be compensated with $50 for completing the survey. This payment will be sent via E-transfer or mailed to the address you provide below, depending on your preference. No receipts are required for reimbursement.

1. **Do you prefer using E transfer or mailed cheque to receive the reimbursement?**
   - ○ **E transfer**
   - ○ **Mailed cheque**

2. **Please enter your VCH email address below, this information will be used to verify employment with VCH and for e-transfer (if indicated as preference):**

3. Following this study, we plan to conduct one-on-one interviews to explore physiotherapist's experiences regarding providing tele-rehabilitation. If you are interested in being contacted, please check the appropriate box below. If contacted, you will be asked to read a new letter of information and sign a new consent form.

- ○ Yes. Please keep my contact information so that I may be contacted to participate in the next phase of the study.
- ○ No. Please do not keep my contact information. I do not wish to be contacted in the future.
